# Age-related acceptability of vaginal self-sampling in cervical cancer screening at two university hospitals: a pilot cross-sectional study

**DOI:** 10.1186/s12889-019-7292-1

**Published:** 2019-07-18

**Authors:** Noely Paula Cristina Lorenzi, Lara Termini, Adhemar Longatto Filho, Maricy Tacla, Lana Maria de Aguiar, Mariana Carmezim Beldi, Edson Santos Ferreira-Filho, Edmund Chada Baracat, José Maria Soares-Júnior

**Affiliations:** 1 Hospital Universitario (HU-USP), Universidade de São Paulo, Sao Paulo, SP Brazil; 2 Center for Translational Investigation in Oncology, Instituto do Câncer do Estado de São Paulo (ICESP), Hospital das Clinicas HCFMUSP, Faculdade de Medicina, Universidade de Sao Paulo, Sao Paulo, Brazil; 3 Molecular Oncology Research Center, Barretos Cancer Hospital/Pio XII Foundation, Barretos, SP Brazil; 4 Department of Pathology, Faculdade de Medicina, Universidade de Sao Paulo, Sao Paulo, Brazil; 5 Research Institute of Life and Health Sciences (ICVS), University of Minho, Braga, Portugal; 6 8ICVS / 3B’s - Associated Laboratory to the Government of Portugal, Braga / Guimarães, Portugal; 7 Disciplina de Ginecologia, Departamento de Obstetrícia e Ginecologia, Hospital das Clinicas HCFMUSP, Faculdade de Medicina, Universidade de Sao Paulo, Sao Paulo, Brazil

**Keywords:** Vaginal self-sampling, Cervical cancer screening, Human papillomavirus

## Abstract

**Background:**

To determine whether age is a barrier against acceptability of cervicovaginal self-sampling in screening for cervical cancer at two gynecology outpatient clinics.

**Methods:**

This is a cross-sectional study involving 116 women over 21 years of age with an abnormal Pap smear. Clinical and laboratorial data were recorded in electronic files. Women received detailed self-collection instructions. After the self-sampling procedure (Evalyn Brush®), women were instructed to answer a questionnaire about vaginal self-sampling acceptability that consisted of seven multiple-choice items. The participants were divided into three age brackets: 21 to 29 years, 30 to 49 years, and 50 years and over. Chi-square, Fischer exact, Kolmogorov-Smirnov and Kruskal-Wallis tests were used.

**Results:**

The analysis of the participants’ perception of the procedure stratified according to age groups showed a decline in the fear of hurting oneself during the procedure as age increased. Most participants reported that it was very easy to understand how to use the self-sampling brush and that it was easy to use it. Most of them were neither embarrassed nor afraid of getting hurt during the procedure. The majority preferred self-sampling to collection by a healthcare professional. The main reason was practicality: the possibility of choosing the place and time for sampling.

**Conclusions:**

The participating women found self-collection simple to understand and easy to accept regardless of age. The younger women indicated more fear and discomfort in self-sampling, which points to the need for attraction strategies that are more appealing to the younger generations.

## Background

Cervical cancer is one of the leading causes of death from cancer in adult women [1]. In Brazil in 2012, there were 17,540 new cases, with an estimated risk of 17 cases for every 100,000 women [2]. Brazilian Health Ministry launched the National Program for the Control of Cervical Cancer to decrease the incidence rate. However, more than 6 years later, the incidence of cervical cancer remained high, with 16,370 cases reported [3]. For example, Amazonas, a state in northern Brazil with many public health issues, has the highest cervical cancer incidence in the country, with 37.1 registered cases per 100,000 women. Even in the state of São Paulo, which has one of the best structured health care systems in Brazil, the incidence is still high with 9.5 cases per 100,000 women [3].

The low socioeconomic status of the population may partially justify the persistence of the problem, for it prevents easy access to healthcare. However, in larger metropolitan areas like São Paulo, cervical cancer still poses a challenge [4] despite the relative nearness to better health care systems. Hence, there must be other factors influencing women’s participation in cervical cancer screening. One such factor might be the very nature of the gynecological examination required for a Pap smear collection: it may be deemed embarrassing and unacceptable by some women [5] because of underlying emotional or psychological issues.

The risk of developing invasive cancer is nearly 3 to 10 times greater in women who do not undergo screening [6, 7]. Thus, measures encouraging women to participate in screening programs indirectly have the potential to save lives and minimize suffering. Furthermore, the attendant decrease in advanced stages of cancer reduces treatment expenditures, thereby optimizing government funds [8]. However, a screening program may be deemed successful only if it covers a large segment of the population [9]. Undermining potential success is the fact that women from lower socioeconomic groups have been less participant than those from more affluent backgrounds [10]. In addition, the participation rate of women in the 25 to 29 age bracket in England has declined recently [5, 11]. This trend may spread to other parts of the world, including Brazil, further decreasing coverage.

The following relevant factors hinder cervical cancer screening: a) lack of infrastructure in some regions; b) hard-to-reach places; c) need for a specialized and well-trained healthcare team; d) follow-up requiring multiple office visits; e) difficulty imposing quality control standards for the procedure, and f) lack of resources to provide the necessary treatment. These deterrents are compelling reasons to seek alternative screening methods [12].

Increasing knowledge of the natural development of the disease and advances in molecular methodology have culminated in new screening methods for detecting the genetic material of human papillomavirus (HPV), specifically high-risk HPV (hrHPV). Molecular tests seem to be a real option in the detection of high-grade lesions in population screening programs given their high negative predictive value and high sensitivity [13]. They have the additional advantage that they can be carried out with vaginal/cervical material collected by the patient herself [14]. Moreover, data from systematic reviews of the literature on the acceptability of self-collection have shown that the method is well accepted [15]. This fact may be used to encourage women to participate in screening programs for cervical cancer [16, 17]. Nonetheless, some studies have shown that only a few women are concerned about their ability to perform self-collection correctly or they are afraid of experiencing some discomfort during the procedure [16, 18].

Vaginal self-collection has the potential to increase the number of women screened for cervical cancer because women who do not undergo screening via the conventional gynecological examination due to fear, shame, functional orthopedic limitations, joint diseases, neurological disorders, obesity, and cultural or religious reasons may agree to self-sampling [19–21]. However, it is unclear to what extent this method is accepted by the overall population in São Paulo. Postulating that acceptability is influenced by age, it is possible to assume that younger patients, being more receptive to new methods and technology, will accept the self-collection procedure more easily. Therefore, this study aimed to determine the influence of age on the acceptability of self-collection at two university hospitals in São Paulo City.

## Methods

### Study design, setting and participants

This is a cross-sectional study with 116 women who were at least 21 years old and were admitted to our colposcopy outpatient clinic because of an abnormal Pap smear. We used a non-probabilistic sampling method (convenience sample), due to the easy access we had to these individuals while attending medical appointments. Patients were prospectively recruited, between 2015 and 2018, and all eligible women were invited to participate as subjects of this research (consecutive recruitment). All patients were aware of the important meaning of their gynecology appointment. These women were examined at the Gynecology Outpatient Clinic at both the Hospital Universitário da Universidade de São Paulo (HU-USP) and the Hospital das Clínicas da Faculdade de Medicina da Universidade de São Paulo (HC-FMUSP).

#### Inclusion criteria

Participants were referred for colposcopy due to the following colpocytological changes (2001 Bethesda System): atypical squamous cells not precluding high-grade lesion (ASC-H); high-grade squamous intraepithelial lesion (HSIL); persistent low-grade squamous intraepithelial lesion (LSIL) or LSIL in immunosuppressed patient; atypical but possibly nonneoplastic glandular cells (AGC); atypical glandular cells favoring neoplasms (AGC-FN); adenocarcinoma in situ (AIS); squamous carcinoma (SQC); and invasive or microinvasive adenocarcinoma.

#### Exclusion criteria

Women under 21 years of age; pregnant women; and those unwilling to participate in the research protocol.

### Ethics consideration

This study was approved by the Ethics Committees of HU-USP (Comitê de Ética em Pesquisa do Hospital Universitário da Universidade de São Paulo) and HC-FMUSP (Comissão de Ética para Análise de Projetos de Pesquisa - CAPPesq) with the registration numbers 38719314.2.3001.0076 and 38719314.2.0000.0068, respectively. Participants were included after signing an informed consent statement.

### Procedure

On the first visit, clinical and epidemiological data were collected from the 116 participants (Table 1). They initially received verbal instructions and a leaflet with illustrations explaining how to carry out the self-collection. Each participant was then directed to a specific and secluded place in the medical office where she could follow the procedure. Self-collection was performed with a sterile Evalyn Brush® (Rovers®, Oss, the Netherlands).

**Table 1:** Descriptive analysis stratified by age, including absolute and relative frequencies and confidence interval of 95% (CI95%)

	≤ 29 years		30–49 years		≥ 50 years	
	N	% (CI95%)	N	% (CI95%)	N	% (CI95%)
Coitus						
≤ *16 years*	20	60.6 (43.6–75.8)	31	45.6 (34.1–57.4)	1	6.7 (0.70–27.2)
*> 16 years*	13	39.4 (24.2–56.4)	37	54.4 (42.6–65.9)	14	93.3 (72.8–99.3)
Parity						
≤ *1 delivery*	21	63.6 (46.6–78.4)	25	36.8 (26.0–48.6)	4	26.7 (9.7–51.7)
*> 2 deliveries*	12	36.4 (21.6–53.4)	43	63.2 (51.4–74.0)	11	73.3 (48.3–90.3)
Lifetime number of sexual partners						
≤ *5 partners*	21	63.6 (46.6–78.4)	36	52.9 (41.2–64.5)	11	73.3 (48.3–90.3)
*> 5 partners*	12	36.4 (21.6–53.4)	32	47.1 (35.5–58.8)	4	26.7 (9.7–51.7)
Contraceptive method						
*Hormonal*	22	66.7 (49.7–80.8)	30	44.1 (32.8–56)	2	13.3 (2.9–36.3)
*Nonhormonal / Does not use it*	11	33.3 (19.2–50.3)	38	55.9 (44–67.2)	13	86.7 (63.7–97.1)
Schooling						
*Illiterate / literate*	15	45.5 (29.4–62.2)	25	36.8 (26–48.6)	10	66.7 (41.6–86)
*Completed high school / college*	18	54.5 (37.8–70.6)	43	63.2 (51.4–74)	5	33.3 (14–58.4)
Religion						
*Catholic*	18	54.5 (37.8–70.6)	46	67.6 (56–77.9)	9	60 (35.3–81.2)
*Non-Catholic*	15	45.5 (29.4–62.2)	22	32.4 (22.1–44)	6	40 (18.8–64.7)
Ethnicity						
*Non-Caucasian*	16	48.5 (32.2–65.1)	31	45.6 (34.1–57.4)	5	33.3 (14.0–58.4)
*Caucasian*	17	51.5 (34.9–67.8)	37	54.4 (42.6–65.9)	10	66.7 (41.6–86.0)
Tobacco						
*No*	26	78.8 (62.8–90.0)	47	69.1 (57.5–79.1)	12	80.0 (55.6–94.0)
*Yes*	7	21.2 (10–37.2)	21	30.9 (20.9–42.5)	3	20.0 (6.0–44.4)
Alcohol						
*No*	18	54.5 (37.8–70.6)	47	69.1 (57.5–79.1)	12	80 (55.6–94)
*Yes*	15	45.5 (29.4–62.2)	21	30.9 (20.9–42.5)	3	20 (6–44.4)
Menopause						
*No*	33	100	68	100	4	26.7 (9.7–51.7)
*Yes*	0	–	0	–	11	73.3 (48.3–90.3)

### Instructions for using the Evalyn brush®

Instructions for self-collection were as follow: “First wash your hands; then take the brush and find a comfortable position (standing or lying down); spread the vaginal lips apart with one hand and, with the other, insert the collector tube in the vagina until the flaps touch the vaginal lips; with one hand, hold the transparent tube and, with the other, push the pink plunger into the vagina. When the brush is in the correct position, you will hear a click; rotate the plunger five times in the same direction. At each rotation, you will hear a click, so you can count the number of rotations; take out the tube carefully and pull the pink plunger until the brush disappears into the tube”.

Once collected, the material was handed over to the health professional, who placed it in a bottle containing cell-preservation liquid for carrying out liquid-based cytology for HrHPV detection. The participants were then invited to answer a questionnaire on the acceptability of self-collection.

### Age-related analysis of groups

Most cervical cancers occur at a reproductive age with incidence peaking between 45 to 50 years of age. In 2014, the United States (US) Food and Drug Administration (FDA) approved an HPV DNA test for primary cervical cancer screening. The US Preventive Services Task Force (USPSTF) recommends that women older than 30 be tested for hrHPV every 5 years [22]. Furthermore, the World Health Organization (WHO) recommends that women aged 30 to 49 years be screened for HPV for protection against cervical cancer [23]. However, in recent years, it has been shown that attendance rates in some European countries have dropped among women aged 25 to 29 [11, 24, 25]. Consequently, we opted for dividing participants into 3 age groups to assess self-collection acceptability as follows: a) 21 to 29 years; b) 30 to 49 years; c) 50 and older.

### Variables

To obtain a detailed report on the participants’ opinion about self-collection, women were invited to answer the Acceptability of Vaginal Self-Collection questionnaire, covering seven topics based on studies of the subject [16, 18, 26–29]. The independent variables and the acceptability of vaginal self-collection questionnaire are synthetized in Table 2.

**Table 2:** Dependent and independent variables used in this study

Dependent variables	Patient options
Understanding how to use the brush was?	a) Very easy
	b) Easy
	c) A bit difficult
	d) Difficult
Using it?	a) Very easy
	b) Easy
	c) A bit difficult
	d) Difficult
How does it feel?	a) Very painful or uncomfortable
	b) Painful or uncomfortable
	c) Not very painful or uncomfortable
	d) Neither painful nor uncomfortable
Were you afraid of hurting yourself?	a) Very afraid;
	b) Afraid;
	c) Not very afraid;
	d) I was not afraid.
If on your pap test you could choose between self-collection and collection conducted by a healthcare professional, which would you choose?	a) Self-collection;
	b) Collection by a healthcare professional;
	c) Either one.
Why would you choose self collected samples?	a) Less pain or discomfort;
	b) Less shame or embarrassment;
	c) Practicality;
	d) Possibility of collecting the sample either at home or at the laboratory;
	e) Fear of not collecting the sample properly;
	f) I wouldn’t - The healthcare professional can do a better job.
Independent variables	Patient options
Ethnicity	a) Caucasian
	b) Non-Caucasian
Religion	a) Roman Catholic
	b) non-Roman Catholic
Schooling	a) Schooling
	b) No schooling
Smoking history	a) Current smoker
	b) Non-smoker
Alcohol consumption	a) Currently
	b) In the past or never
Age at first intercourse	a) Early: ≤ 16 years
	b) Late: ≥ 16 years);
Number of vaginal deliveries or caesarean sections	a) ≤ 1
	b) ≥ 2
Number of sexual partners	a) ≤ 5
	b) ≥ 6
Use of contraceptive	a) Hormonal
	b) Non-hormonal

### Statistical analysis

Quantitative analysis of the study data produced frequency tables for qualitative variables with a 95% confidence interval (95% CI). Measures of central tendency and dispersion were calculated for the quantitative variables. Boxplots were used to explore age distribution according to the different outcomes.

The chi-square test or the Fischer exact test was utilized for comparing group percentages in the case of categorical variables. The Kolmogorov-Smirnov and Shapiro-Wilk tests were used to assess the normality of the age variable. The Kruskal-Wallis test was used for testing the hypothesis that age was similarly distributed among the different outcome categories of the 1 through 6 issues of the questionnaire.

All tests used a bidirectional α of 0.05 and a 95% CI. Calculations were made with the IBM SPSS 25 (Statistical Package for the Social Sciences) and Excel 2010® (Microsoft Office) software programs.

## Results

A total of 116 women whose mean age was 36.2 ± 10.4 years was assessed. Descriptive analysis of the participants by age bracket yielded the following results: a) ≤ 29 years of age, 33 (28.4%) women, (95%CI, 20.8–37.1%); b) 30–49 years of age, 68 (58%) women (95%CI, 49.5–67.3%); c) ≥ 50 years of age, 15 (12.90%) women, (95%CI, 7.8–19.9%). Table 1 displays the sociodemographic variables. In all three groups, there was a significant difference between first intercourse at an early age (≤ 16 years) and at a late age (> 16 years). The highest proportion (93.3%; 95%CI, 72.8–99.3%) of the latter was found in the over 50 age bracket. This very same bracket had the largest number of postmenopausal women (73.3%; 95%CI, 48.3–90.3%). In the 29 or under 29 age bracket, there was a case of a woman with premature ovarian failure. The remaining variables were homogeneous in terms of age categories (95%CI) with no statistically significant differences (Table 1).

Table 3 shows the summarized data on the acceptability of self-collection (A - Understanding how to use the self-collection brush, B - Using the self-collection brush, C - Discomfort or pain when using the self-collection brush, D - Embarrassment or shame when using the self-collection brush, E - Fear of hurting oneself when using the self-collection brush, and F - Self-collection vs. collection by a health professional). Most women reported that questions A (Understanding how to use the self-collection brush) and B (Using the self-collection brush) were easy or very easy to understand; there were no significant statistical differences among the age groups. Most of the women also stated their preference for self-collection (76.70%; 95% CI, 68.40–83.70%). For a small percentage of women (12.9%; 95% CI, 7.8–19.9%), collection performed by a health professional was their choice. The remaining women (10.3%; 95% CI, 5.8–16.9%) declared their indifference to the choice of procedure. Answers to the collection questions did not differ significantly among the age groups. Irrespective of age group, women indicated lack of shame or embarrassment in question D (Embarrassment or shame when using the self-collection brush).

**Table 3:** Analysis of women’s perceptions of the self-collection brush stratified by age, including absolute and relative frequencies and descriptive level (*p*-value)

	≤ 29 years		30–49 years		≥ 50 years		Total	p
	N	%	N	%	N	%		
1. Understanding how to use the self-collection brush								
*Very easy*	16	28.1	36	63.2	5	8.8	57	0.591
*Easy*	17	29.3	31	53.4	10	17.2	58	
*A bit difficult*	0	0.0	1	100.0	0	0.0	1	
2. Using the self-collection brush								
*Very easy*	9	22.5	27	67.5	4	10.0	40	0.378
*Easy*	22	30.6	40	55.6	10	13.9	72	
*A bit difficult*	2	50.0	1	25.0	1	25.0	4	
3. Discomfort or pain when using the self-collection brush								
*Painful, uncomfortable*	0	0.0	6	100.0	0	0.0	6	0.080
*Not very painful, uncomfortable*	11	44.0	13	52.0	1	4.0	25	
*Not painful, uncomfortable l*	22	25.9	49	57.6	14	16.5	85	
4. Embarrassed or ashamed when using the self-collection brush								
*Embarrassed/ashamed*	1	50.0	1	50.0	0	0.0	2	0.427
*Not very embarrassed/ashamed*	9	37.5	14	58.3	1	4.2	24	
*Not embarrassed/ashamed*	23	25.6	53	58.9	14	15.6	90	
5. Fear of hurting oneself when using the self-collection brush								
*Very afraid*	1	100.0	0	0.0	0	0.0	1	0.025
*Afraid*	5	71.4	2	28.6	0	0.0	7	
*Not very afraid*	7	33.3	9	42.9	5	23.8	21	
*Not afraid*	20	23.0	57	65.5	10	11.5	87	
6. Self-collection vs. collection by a health professional								
*Self-collection*	27	30.3	49	55.1	13	14.6	89	0.769
*Health professional*	4	26.7	10	66.7	1	6.7	15	
*Indifferent*	2	16.7	9	75.0	1	8.3	12	
Total	33	28.4	68	58.6	15	12.9	116	

There was a statistically significant difference in responses to question E (Fear of hurting oneself when using the self-collection brush) as to age: fear decreased as age increased. Only one woman in the 29 or under 29 age bracket indicated much fear. Of those who registered fear, 71.4% were 29 or younger. Of those who indicated little fear, the largest percentage, 42.9%, was found in the 30 to 39 age category and 11.5% in the 50 or older age bracket (*p* = 0.025). Question C (Discomfort or pain when using the self-collection brush) revealed the same tendency; however, it was not statistically significant (*p* = 0.080).

Table 4 shows the reasons women opted for self-collection or a health professional. None of the items differed significantly in terms of age brackets. However, among those who chose self-collection, practicality was the preferred variable for all three age groups, followed by less embarrassment for both end brackets, and finally, largely for the middle bracket, the possibility of performing the procedure at home, at the laboratory, or at a basic health facility (BHF).

**Table 4:** Reasons women opted for self-collection or a health professional, stratified by age, including absolute and relative frequencies and a confidence interval of 95% (CI 95%)

	≤ 29 years		30–49 years		≥ 50 years	
	N	% (CI95%)	N	% (CI 95%)	N	% (CI 95%)
Less pain or discomfort^a^						
No	18	58.1 (40.6–74.1)	30	50.8 (38.3–63.3)	9	64.3 (38.5–84.9)
Yes	13	41.9 (25.9–59.4)	29	49.2 (36.7–61.7)	5	35.7 (15.1–61.5)
Less shame or embarrassment^a^						
No	15	48.4 (31.6–65.5)	38	64.4 (51.7–75.7)	6	42.9 (20.3–68.1)
Yes	16	51.6 (34.5–68.4)	21	35.6 (24.3–48.3)	8	57.1 (31.9–79.7)
Practicality^a^						
No	12	38.7 (23.2–56.2)	24	40.7 (28.8–53.4)	3	21.4 (6.4–46.9)
Yes	19	61.3 (43.8–76.8)	35	59.3 (46.6–71.2)	11	78.6 (53.1–93.6)
Self-collection at home/BHF/Lab^a^						
No	21	67.7 (50.3–82.1)	28	47.5 (35.1–60.1)	8	57.1 (31.9–79.7)
Yes	10	32.3 (17.9–49.7)	31	52.5 (39.9–64.9)	6	42.9 (20.3–68.1)
Afraid of not collecting it correctly^b^						
No	29	93.5 (80.9–98.6)	54	91.5 (82.4–96.7)	13	92.9 (71.2–99.2)
Yes	2	6.5 (1.4–19.1)	5	8.5 (3.3–17.6)	1	7.1 (0.8–28.8)
The health professional can do it better^b^						
No	29	93.5 (80.9–98.6)	53	88.3 (78.5–94.6)	14	100
Yes	2	6.5 (1.4–19.1)	7	11.7 (5.4–21.5)	0	–

^a^ Variables checked by the women who opted for self-collection

^b^ Variables checked by the women who opted for a health professional

Table 5 shows the outcomes of women’s perception of the use of a self-collection brush. The results, which point to some discomfort or pain in using the brush, are heterogeneous in terms of age distribution (mean age and median age); however, not significantly so.

**Table 5:** Mean age and median age of the women checking the variables under the categories related to the self-collection brush, including measurements of central tendency, position, and dispersion

	Mean	SD	Median	P25	P75	*p*-value
1.Understanding how to use the self-collection brush						
*Very easy*	35	10	34	28	40	0.396
*Easy*	37	11	36	27	42	
*A bit difficult*	44	–	44	44	44	
*Difficult*	–	–	–	–	–	
2. Using the self-collection brush						
*Very easy*	36	9	34	30	41	0.871
*Easy*	37	11	36	27	42	
*A bit difficult*	34	11	30	28	41	
*Difficult*	–	–	–	–	–	
3. Discomfort or pain when using the self-collection brush						
*Very painful, uncomfortable*	–	–	–	–	–	
*Painful, uncomfortable*	39	6	37	36	42	
*Not very painful, uncomfortable*	33	9	31	26	35	0.081
*Not painful, uncomfortable*	37	11	36	29	42	
4. Embarrassed or ashamed when using the self-collection brush						
*Very embarrassed/ashamed*	–	–	–	–	–	
*Embarrassed / ashamed*	32	7	32	27	37	0.308
*Not very embarrassed / ashamed*	33	8	32	27	39	
*Not embarrassed / ashamed*	37	11	36	29	42	
5. Afraid of hurting oneself when using the self-collection brush						
*Very afraid*	26	–	26	26	26	0.167
*Afraid*	30	5	27	27	31	
*Not very afraid*	37	13	35	25	42	
*Not afraid*	37	10	35	30	42	
6. Self-collection vs. collection by a health professional						
*Self-collection*	36	11	34	27	41	0.218
*Health professional*	34	7	32	27	37	
*Indifferent*	40	10	42	33	46	

**Fig. 1:**
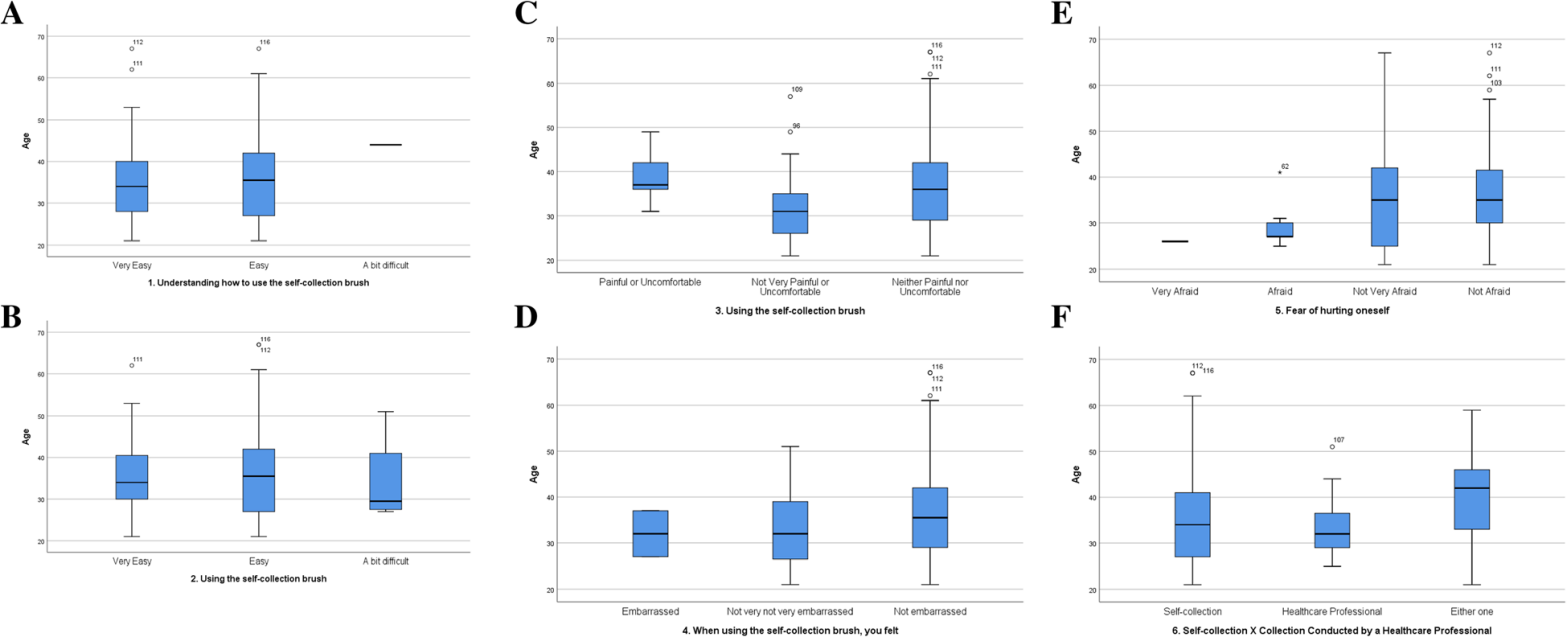
A graphical representation of the answers from the acceptability questionnaire stratified by age: **a** age distribution of the item Understanding how to use the self-collection brush; **b** age distribution of the item Using the self-collection brush; **c** age distribution of the item Discomfort or pain when using the self-collection brush; **d** age distribution of the item Embarrassed or ashamed when using the self-collection brush; **e** age distribution of the item Fear of hurting oneself when using the self-collection brush; **f** age distribution of the item Self-collection vs. collection by a health professional

Fig. 1 displays the results of each age bracket corresponding to the A to F answers on the questionnaire.

In the multivariate analysis of the influence of the sociodemographic data on the acceptability questionnaire, the outcome *Understanding how to use the self- collection brush* was found significantly associated with the following two variables: a) *parity,* with 62% of the women in the *≤1 delivery* category reporting it was very easy to use the self-collection brush (*p* = 0.011), and b) *alcohol*, with 64.1% of the women admitting the very same thing (*p* = 0.045). There were no differences with respect to the other items.

## Discussion

Wide acceptance of the self-collection procedure requires that it be essentially adequate and easy to understand as with any new method. However, for an even more inclusive adoption, it should also appeal to the women who have previous experience with the usual method, i.e., screening through a physician-conducted speculum examination. If women are comfortable with the conventional procedure, changing methods is viewed negatively. On the other hand, if there is a desire for change stemming from cultural, social, or emotional reasons or because of shame or fear during the cervicovaginal collection, self-sampling is a welcome change [30]. In our study, which was conducted at university hospitals addressing secondary and tertiary health care, most of the participants, irrespective of age bracket, accepted and easily understood the self-sampling device and procedure. Of these, a large percentage had previous experience with conventional screening; nonetheless, they opted for self-screening as their method of choice. Still, one fifth of subjects preferred health provider collection or were indifferent. Numerous studies in several countries report on the influence of the cultural, emotional, and sociodemographic aspects of different ethnic groups as well as their personal convictions in accepting vaginal self-sampling [15, 27, 31–35]. Notwithstanding the influence of such factors, recent meta-analysis, comprising 37 studies and involving 18.516 women in 24 countries in 5 continents, reinforced acceptance of and preference for self-collection in the study communities [36]. In the Brazilian population, our study shows the same acceptance.

In our study we used a leaflet with illustrations and a FAQ (Frequently Asked Questions) section to guide the patient through the self-collection procedure. A physician was also available to answer any questions the patients might have. This was possibly one of the resources which helped understand and facilitate the use of the method. In other studies, women also indicated their preference for self-collection as long as they were adequately instructed on how to collect the samples [16, 37, 38]. Fear of or apprehension about not carrying out the procedure adequately could be a factor limiting acceptance, as reported in other studies [39, 40]. Such a negative feeling was not confirmed, for most of the large number of participants they included indicated satisfaction [41–43]. Nor did the majority in our study express apprehension.

Discomfort or pain when using the self-collection brush was another concern. In fact, in a study conducted in a low-resource setting showing wide self-collection acceptability, nearly half of the nonparticipating women interviewed expressed their fear of getting hurt while using the self-sampling brush and almost a quarter were afraid to drop the brush during the procedure [33]. Study participants reported that their main concern with regard to screening was the possibility of being diagnosed with precancer or cancer, of having to take some time off work or leave home for the procedure, or of not knowing whether screening was really necessary given the absence of signs or symptoms of a disease [33].

In this sense, the participants in the oldest age bracket had previous experience with cervical cancer screening programs as well as the knowledge that participation was necessary as a preventive factor; moreover, they could plan and make time available for a consultation and transportation when necessary. These aspects may be relevant factors in the study results [33]. It should be emphasized that such factors must be overcome to gain women’s confidence and receptivity and thus increase the number of participants in the screening programs [28]. These findings are particularly important because screening women with an HPV-DNA test just once at 35 years of age has the potential to reduce a lifetime risk of cervical cancer by 36% [44].

In our study, it was the youngest participants who indicated the greatest fear and discomfort with respect to self-collection. This agrees with other publications, which mention that the young participants were more skeptical and suspicious than the older women [25, 45]. Skepticism that alters with age points to a need for different screening supports for each age bracket. All should have in common, though, clear step-by-step guidelines for self-collection along with educational messages that are also appealing to the younger generations. Furthermore, it is important to adapt the language used to these younger women. Use of smartphone apps and text messaging with more concise language [46] may also increase acceptance of self-collection.

Our study, in agreement with others, did not discern any specific cultural or religious barriers to self-sampling [32, 40]. However, a study in the UK found that Muslim women were reluctant to try the self-collection approach [16] much the same as Mexican indigenous women [47].

One could understand that a correlation between alcohol consumption and a very easy understanding of the self-collection procedure and use of the brush might be due to the fact that mild consumption of alcohol can reduce anxiety and fear brought by life’s new challenges [48]. However, we recommend that the difference between the *very easy* and *easy* answers should be considered carefully because these two variables may not be sensitive enough to establish a clear boundary between them.

Self-sampling for the detection of hrHPV types has already been implemented in many countries with the purpose of increasing participation in cervical cancer screening and, consequently, improve outcomes [49]. One such example is the Netherlands, the first country to offer women the option to self-collect samples for HPV testing [50–52].

Our study has some limitations in scope because there was a restrict number of participants and further studies including the general community are needed to clarify whether age is a barrier. Also, instructions should be adapted to increase acceptability of the method mostly by the younger population. On the other hand, the fact that this study was carried out with a population experienced in Pap smear collection by a health professional is its strong point. Finally, our results suggest Brazilian women are receptive to vaginal self-sampling. Nevertheless, since there are differences among age brackets, each group should receive age-appropriate instructions.

## Conclusion

The study participants found the self-collection procedure easy to accept and understand irrespective of age.

## Data Availability

The datasets used and analyzed during the current study are available from the corresponding author on reasonable request.

## Abbreviations

95%CI: 95% confidence interval
AGC: Atypical but possibly nonneoplastic glandular cells
AGC-FN: Atypical glandular cells favoring neoplasms
AIS: Adenocarcinoma in situ
ASC-H: Atypical squamous cells not precluding high-grade lesion
BHF: Basic health facility
CAPPesq: Comissão de Ética para Análise de Projetos de Pesquisa
DNA: Deoxyribonucleic acid
FAQ: Frequently asked questions
FDA: Food and Drug Administration
HC-FMUSP: Hospital das Clínicas da Faculdade de Medicina da Universidade de São Paulo
HPV: Human papillomavirus
hrHPV: High-risk HPV
HSIL: High-grade squamous intraepithelial lesion
HU-USP: Hospital Universitário da Universidade de São Paulo
LSIL: Low-grade squamous intraepithelial lesion
SPSS: Statistical Package for the Social Sciences
SQC: Squamous carcinoma
US: United states
USPSTF: US Preventive Services Task Force
WHO: World Health Organization
